# Protein O-glucosyltransferase 1 overexpression downregulates p16 in BT474 human breast cancer cells

**DOI:** 10.3892/ol.2014.2197

**Published:** 2014-05-28

**Authors:** GANG JIN, ZHIGANG CAO, XILIN SUN, KAI WANG, TAO HUANG, BAOZHONG SHEN

**Affiliations:** 1 Department of Medical Imaging, The Fourth Affiliated Hospital of Harbin Medical University, Harbin, Heilongjiang 150001, P.R. China; 2 The No. 211 Hospital of the People’s Liberation Army, Harbin, Heilongjiang 150086, P.R. China; 3 The Second Affiliated Hospital of Harbin Medical University, Harbin, Heilongjiang 150001, P.R. China

**Keywords:** protein O-glucosyltransferase 1, transforming growth factor β1, BT474 human breast cancer cells, p16, Smad3

## Abstract

Protein O-glucosyltransferase 1 (POGLUT1) is a novel gene that was initially isolated and identified from the bone marrow cells of patients with myelodysplastic syndrome/acute myeloid leukemia. Previous findings have suggested that POGLUT1 promotes the proliferation of U937 human tissue lymphoma cells. Furthermore, POGLUT1 has been identified in other tissues, including the mammary glands, lymph nodes, intestine, liver and spleen. In the present study, in order to investigate the function and target of POGLUT1 in BT474 breast cancer cells, the effect of POGLUT1 on cell proliferation, differentiation, apoptosis and key proteins in the transforming growth factor (TGF)-β1 signaling pathway was investigated in BT474 cells. The overexpression of POGLUT1 in the presence of TGF-β1 was found to significantly enhance cell viability. Flow cytometric and quantitative polymerase chain reaction analyses revealed that POGLUT1 had an effect on the cell cycle and inhibited the TGF-β1-induced transcriptional upregulation of p16, a major cyclin-dependent kinase inhibitor (CDKI). Furthermore, phosphorylated (p)-Smad3, which has a key role in mediating the TGF-β antiproliferative response, was greatly inhibited by exogenous POGLUT1, suggesting a role for POGLUT1 in the TGF-β1-mediated signaling pathway in the BT474 cell cycle. However, no significant changes were observed in the expression of other CDKIs or in cell apoptosis. The findings of the present study show that the increase in BT474 cell viabilty induced by POGLUT1 is associated with POGLUT1-induced inhibition of the transcriptional upregulation of p16 by TGF-β1, which may be a result of the inhibition of p-Smad3.

## Introduction

Protein O-Glucosyltransferase 1 (POGLUT1), also known as Rumi, MDSRP or hCLP46(1–4), was initially identified in CD34^+^ cells of patients with acute myeloid leukemia that had transformed from myelodysplastic syndrome. POGLUT1 contains a highly conserved domain termed CAP10, as well as an endoplasmic reticulum retention signal motif, KTEL, at the C-terminus and a hydrophobic signal peptide at its N-terminus (5,6). Previous studies have reported that BT474 human breast cancer cell growth increases in response to POGLUT1 overexpression due to POGLUT1-induced inhibition of transforming growth factor β1 (TGF-β1)-mediated induction of INK4a gene expression (7,8). TGF-β1 is a multifunctional cytokine with a central role in the regulation of numerous biological processes, including cell proliferation, differentiation and the modulation of immune responses (9). TGF-β1 induces its various effects through serine/threonine kinase transmembrane receptors and induces signaling from receptors to the nucleus mediated through the phosphorylation of cytoplasmic effector molecules of the Smad protein family (10). Phosphorylated (p)-Smad2 and Smad3 form heteromeric complexes with Smad4, which are then translocated to the nucleus where they function as transcription factors (11–13). TGF-β1 signaling has been reported to increase during the inhibition of cell cycle progression, through activating cyclin-dependent kinase inhibitors (CDKIs) and inactivating c-Myc (14–16). A number of studies have investigated the TGF-β1 signaling blockade inhibiting parathyroid hormone-related protein secretion in breast cancer cells and bone metastases development, as well as the regulatory role of TGF-β1 in gastric cancer cell proliferation and differentiation (17–19).

POGLUT1 may have an important role in cellular self-renewal and the development of various normal and malignant tumor cells. Thus, investigations into the mechanism, interacting molecules and regulation of POGLUT1 in tumor cells are required, particularly in breast cancer which affects numerous females worldwide. This may lead to an enhanced understanding of human breast cancer occurrence and development.

It has been demonstrated that POGLUT1 stimulates the proliferation of U937 human lymphoma cells and inhibits the TGF-β-induced inhibition of U937 cell growth, suggesting that POGLUT1 may be a cytokine which promotes and sustains tumor cell malignant transformation (5). TGF-β activates proteins in the Smad family through a membrane receptor, and activated Smad proteins translocate from the cytoplasm to the nucleus, to enhance the expression of the p16 and p15 target genes (20). In cell cycle regulation, CDKIs, CDKs and cyclin D, the cell cycle protein, form a dynamically balanced system (21–23). POGLUT1 may either downregulate the transcription of the p16 and p15 genes or accelerate the degradation of the p16 and p15 proteins through activating the intracellular proteolytic system.

The present study aimed to investigate the mechanism and signal through which POGLUT1 antagonizes TGF-β1-induced p16 gene expression. In order to investigate the role of POGLUT1 in tumor cell proliferation, a recombinant, Myc-labeled retroviral vector, babe-puro-POGLUT1-Myc, was constructed and transduced into BT474 human breast ductal adenocarcinoma cells to induce exogenous POGLUT1 overexpression. The present study analyzed whether the POGLUT1 gene was capable of antagonizing the activity of TGF-β1, an important inhibitory factor in cell proliferation, thus promoting BT474 cell proliferation. The present study aimed to elucidate the targets of the signaling pathway through which the POGLUT1 gene regulates TGF-β1, Smad3 and p16 activity.

## Materials and methods

### Cell culture and transfection

All of the cells were purchased from American Type Culture Collection (Manassas, VA, USA). BT474 cells were grown in RPMI-1640 (Invitrogen Life Technologies, Carlsbad, CA, USA) supplemented with 10% fetal bovine serum (FBS; Invitrogen Life Technologies) and 293T cells were cultured in Dulbecco’s modified Eagle’s medium (Invitrogen Life Technologies) with 10% FBS. Cells were maintained at 37°C in humidified conditions containing 5% CO_2_. Plasmid transfection was performed using Lipofectamine^®^ 2000 (Invitrogen Life Technologies) according to the manufacturer’s instructions.

### Recombinant retrovirus generation and infection procedure

In order to transfer the POGLUT1 gene into the BT474 cells, a retroviral vector expressing POGLUT1 was constructed. The plasmid vector pcDNA4/POGLUT1-myc contained a POGLUT1-myc-tagged fusion protein for immunodetection. This construct was used to generate a gene-transfer retroviral vector. The POGLUT1-myc cassette amplified by polymerase chain reaction (PCR) and subcloned into the pBabe-puro plasmid (Addgene, Cambridge, MA, USA) using *Bam*HI and *Sal*I sites built into the primers. The primer sequences were as follows: Forward, 5′-ATCCTCGAGCGTAGTTCAGTTTTCAA-3′ and reverse, 5′-ATCGTCGACCTACAGATCCTCTTCTGAGAT-3′. The recombinant retrovirus vector pBabe-POGLUT1-Myc (pBaPM) was identified using sequencing.

To generate a high-titer recombinant retrovirus, pBaPM, pVSV and pHIT60 were contransfected into 293T cells using Lipofectamine 2000. The virus stocks were collected 72 h after transfection then filtered through a 0.45-μm filter and frozen at below −70°C. BT474 human breast cancer cells were inoculated in 96-well culture plates and divided into four groups: pBaPM retrovirus with TGF-β1 group; pBabe blank plasmid without TGF-β1; pBabe blank plasmid with TGF-β1; and blank control group. BT474 cells were also infected with retrovirus stocks for 6 h, and washed and cultured in fresh complete medium, with or without TGF-β1.

### Cell proliferation assay

A colorimetric assay using MTT (Sigma-Aldrich, St. Louis, MO, USA) was performed to assess cell growth and proliferation. In brief, the BT474 cells were inoculated on 96-well culture plates with 1×10^4^ cells/well and 100 μl culture medium per well. TGF-β1 (R&D Systems, Minneapolis, MN, USA) was added to the cells at a final concentration of 100 pg/ml. Fresh medium containing 10% MTT (5 mg/ml stock) was added to each well 72 h after infection. Plates were incubated at 37°C for 3 h then 300 μl dimethyl sulfoxide (Sigma-Aldrich) was added to each well and shaken at room temperature for 10 min to dissolve the intracellular MTT formazan crystals. Absorbance was then measured at 560 nm using an enzyme microplate reader (SpectraMax® M5e; MDS Analytical Technologies, Sunnyvale, CA, USA). Experiments were performed in triplicate and repeated at least twice.

### Western blot analysis

BT474 cells were infected with pBaPM and treated with TGF-β1 (100 pmol/ml). After 48 h, the BT474 cells were incubated in lysis buffer [150 mM NaCl, 1% NP40, 1 mM EDTA, 5 mM benzamidine, 50 mM NaF and 20 mM Tris-HCl (pH 7.6)]. Cell suspensions were vortexed briefly and the protein concentration was determined using the DC Protein Assay (500–0112; Bio-Rad, Hercules, CA, USA) according to the manufacturer’s instructions. Whole cell lysates containing 50 μg total protein were boiled for 5 min in 1× SDS buffer (Takara Bio, Inc., Shiga, Japan), resolved using 10% SDS-PAGE and transferred to nitrocellulose membranes. The membranes were blocked with TBST buffer [0.1 M Tris (pH 7.5), 0.9% NaCl and 0.05% Tween-20 containing 10% non-fat milk powder], then incubated with the following primary monoclonal antibodies: Goat anti-human p16, rabbit anti-human Smad3 and mouse anti-human p-Smad3 (Abcam Cambridge, MA, USA). Membranes were then incubated with anti-goat (rabbit or mouse) horseradish peroxidase-conjugated polyclonal antibodies (Santa Cruz Biotechnology, Inc., Santa Cruz, CA, USA). Immunoreactive proteins were detected using an enhanced chemiluminescence western blotting detection system (WesternBreeze® Chromogenic Kits; Invitrogen Life Technologies). β-actin was used as an endogenous control.

### Fluorescence quantitative PCR (fqPCR) analysis

BT474 cells were infected with pBaPM and treated with TGF-β1 (100 pmol/ml). The cells were collected 48 h after retrovirus infection. Total RNA was extracted using TRIzol^®^ reagent (Invitrogen Life Technologies) according to the manufacturer’s instructions. Complementary (c)DNA was synthesized using SuperScript^®^ II reverse transcriptase (Invitrogen Life Technologies). fqPCR analysis was performed using SYBR^®^ Green (Invitrogen Life Technologies) on an Applied Biosystems 7900HT system (Applied Biosystems, Foster City, CA, USA) to detect POGLUT1 gene transcription. GAPDH expression was used as an endogenous control. The primer sequeneces were as follows: Forward, 5′-GAT ATC ATG TAT CCT GCT TG-3′ and reverse, 5′-TTT TCC ATG GCC ACT GTG GTC-3′ for POGLUT1; and forward, 5′-GGA AGG TGA AGG TCG GAG TC-3′ and reverse, 5′-CGT TCT CAG CCT TGA CGG T-3′ for GAPDH.

The cDNA from the infected BT474 cells was also used to analyze p16 gene expression using fqPCR with TaqMan^®^ probes and the sequences were as follows: p16-F, 5′-CAT AGA TGC CGC GGA AGG-3′; p16-R, 5′-AAG TTT CCC GAG GTT TCT CAG A-3′; and p16-T, 5′FAM-CCT CAG ACA TCC CCG ATT GAA AGA-3′TAMRA.

### Statistical analysis

Statistical analysis was performed using SPSS, version 16.0 (SPSS, Inc., Chicago, IL, USA). MTT assay data were pooled and averaged. The statistical significance of the differences between the control and target data sets was determined using independent sample t-tests. P<0.05 was considered to indicate a statistically significant difference.

## Results

### Cell proliferation assay

The BT474 cells were analyzed using an enzyme microplate reader within 96 h of retrovirus infection, and the data were analyzed to generate a cell growth curve. The BT474 cells that were infected with a blank control plasmid with no TGF-β1 treatment, as well as those that were infected with the pBaPM retrovirus and treated with TGF-β1, grew well. No significant difference (P>0.05) in OD_570_ value was observed between the two groups of cells. However, the BT474 cells that were infected with the blank plasmid and treated with TGF-β1 exhibited a lower OD_570_ value compared with those in the other two groups, between 72 h and 96 h after infection. For example, after 72 h, the OD_570_ values of the cells in the pBaPM retrovirus with TGF-β1 group and the blank plasmid without TGF-β1 group were 0.83 and 0.75, respectively, compared with 0.51 in the cells in the blank plasmid with TGF-β1 group (P<0.01). Moreover, after 96 h, the OD_570_ values of the cells in the pBaPM retrovirus with TGF-β1 group and the blank plasmid without TGF-β1 group were 0.89 and 0.85, respectively, compared with 0.70 in the cells in the blank plasmid with TGF-β1 group (P<0.01; Table I). These findings suggest that TGF-β1 inhibits the proliferation of BT474 cells and that POGLUT1 overexpression promotes the growth of BT474 cells.

### POGLUT1 expression in infected BT474 cells

**Figure 1:**
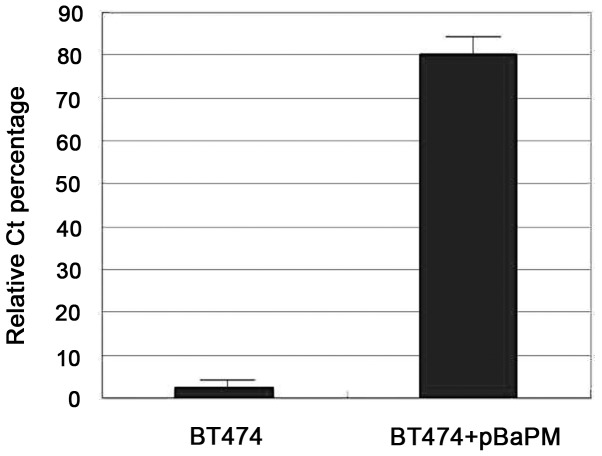
POGLUT1 expression detected using fluorescence quantitative polymerase chain reaction analysis. POGLUT1 expression was increased in the BT474 cells following pBaPM infection, while little endogenous POGLUT1 expression was observed in the wild-type BT474 cells. The relative Ct value of the BT474+pBaPM cells was ~80.1%, which was normalized using the housekeeping gene GAPDH. POGLUT1, protein O-glucosyltransferase; pBaPM, pBabe-POGLUT1-myc; Ct, cycle threshold.

**Figure 2:**
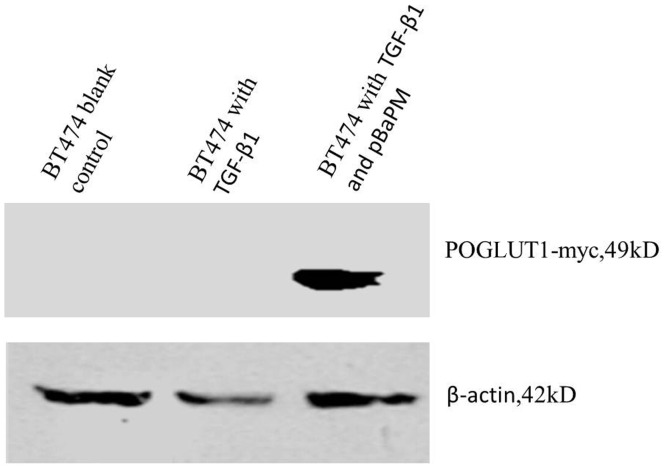
Anti-myc staining representing POGLUT1 expression in BT474 cells following pBaPM infection, to assess the viability of recombinant retrovirus pBaPM in BT474 cells, detected using western blot analysis. No POGLUT1-myc expression was observed in the BT474 cells without or with TGF-β1 treatment; however, abundant expression was found in the BT474 cells infected with pBaPM and treated with TGF-β1. These findings suggest good expression viability of pBaPM. POGLUT1, protein O-glucosyltransferase; pBaPM, pBabe-POGLUT1-myc; TGF-β, transforming growth factor β.

BT474 cells were collected 48 h after retrovirus infection and first-strand cDNA was synthesized using reverse transcription. fqPCR analysis was used to amplify the POGLUT1 gene using SYBR Green. The melting curve for POGLUT1 amplification showed good specificity. fqPCR analysis revealed that the expression of POGLUT1 in the BT474 cells infected with the pBaPM retrovirus was increased compared with the control BT474 cells, where little POGLUT1 expression was observed (Fig. 1). Furthermore, western blot analysis revealed a specific band at ~49 kDa for the POGLUT1-myc protein, while endogenous β-actin showed a band at ~42 kDa (Fig. 2).

### Detection of p16 expression in the infected BT474 cells

p16 expression was found to be increased in the presence of TGF-β1 compared with the untreated BT474 cells (P<0.05). Following pBaPM retrovirus infection, exogenous POGLUT1 was observed to be overexpressed and p16 expression was found to be decreased in the absence of TGF-β1 to a lower degree compared with the control cells (P<0.05). Moreover, in the infected BT474 cells, p16 expression was found to be reduced to a greater degree in the presence of TGF-β1 compared with the control cells (P<0.01). Using the untreated BT474 cells as the baseline, the relative quantity (RQ) value of p16 gene expression was found to be ~76.13*-*fold lower in the POGLUT1+TGF-β1 BT474 cells compared with the TGF-β1-stimulated BT474 cells (RQ=0.062 vs. 4.72; P<0.01), while the RQ value of p16 expression was found to be significantly decreased ~8.92-fold lower in the BT474 cells overexpressing POGLUT1 compared with the TGF-β1-stimulated BT474 cells (RQ=0.53 vs. 4.72; P<0.01) (Table II).

### Effect of POGLUT1 overexpression on p16 protein expression

**Figure 3:**
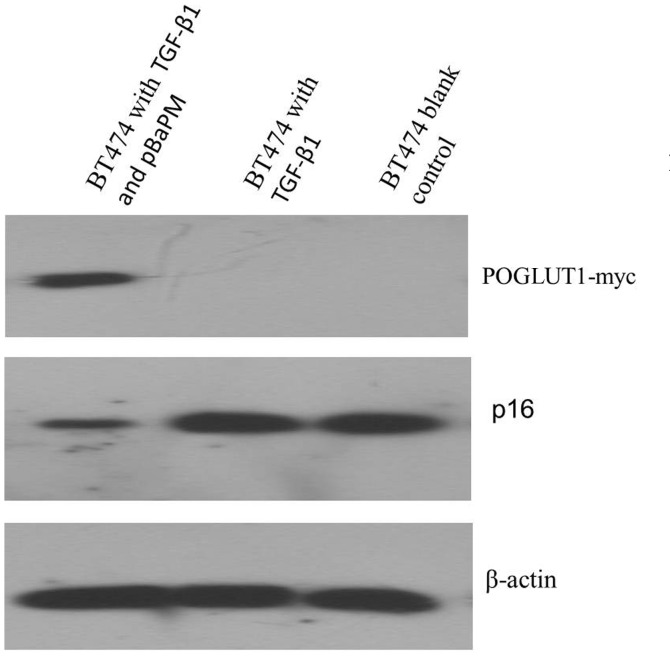
POGLUT1 overexpression represses p16 expression in BT474 cells with TGF-β1. p16 is a cyclin-dependent kinase inhibitor and overexpression of p16 may inhibit cellular proliferation. TGF-β, transforming growth factor β; pBaPM, pBabe-POGLUT1-myc; POGLUT1, protein O-glucosyltransferase.

BT474 cells were infected with the pBaPM retrovirus, cultured in the presence of TGF-β1 for 48 h and collected for the analysis of p16 protein expression using western blot analysis. p16 protein expression was observed in the BT474 cells treated with TGF-β1, as well as the 293T positive control cells. However, very low p16 protein expression was detected in the POGLUT1-overexpressing BT474 cells that were treated with TGF-β1 (Fig. 3).

### Effect of POGLUT1 overexpression on p-Smad3 protein expression

**Figure 4:**
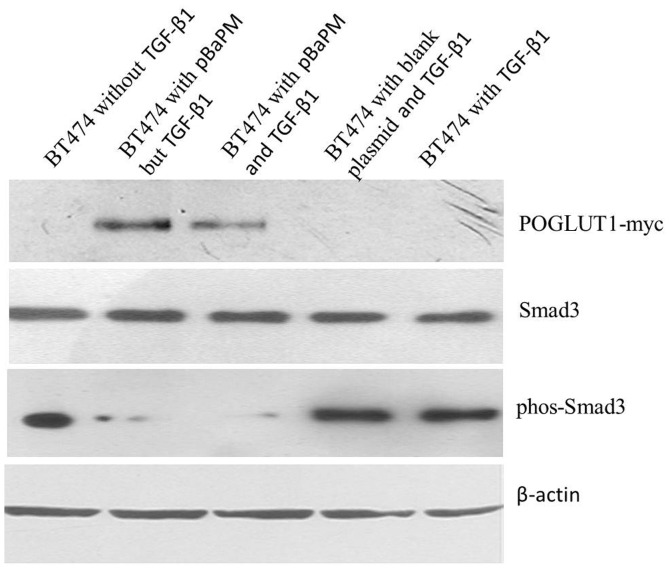
POGLUT1 overexpression inhibits the protein expression of phos-Smad3 in BT474 cells. Smad3 and β-actin proteins were expressed to the same extent in the POGLUT1 overexpression group, blank plasmid group and control group. phos-Smad3 expression was increased in the two control groups and decreased in the POGLUT1 overexpression group with TGF-β1. TGF-β, transforming growth factor β; pBaPM, pBabe-POGLUT1-myc; POGLUT1, protein O-glucosyltransferase; phos, phosphorylated.

The infected BT474 cells were cultured with TGF-β1 for 48 h, then collected for Smad3 and p-Smad3 protein detection using western blot analysis. The overexpression of POGLUT1 was found to inhibit the expression of the p-Smad3 protein in the BT474 cells. The Smad3 protein and the internal control β-actin were observed to be expressed to the same extent in the POGLUT1 overexpression group, blank plasmid group and control group. However, p-Smad3 expression was found to be markedly increased in the two control groups and markedly decreased in the POGLUT1 overexpression group (Fig. 4).

## Discussion

In the present study, pBabe-POGLUT1-myc(pBaPM) retrovirus was recombined, which overexpresses exogenous POGLUT1 in human breast cancer BT474 cells. In addition, the overexpression of exogenous POGLUT1 may repress p16 expression and inhibit the p-Smad3 protein expression in the presence of TGF-β1. First, the recombinant retrovirus vector pBabe-puro-POGLUT1-Myc was constructed *in vitro*, then transformed and packaged into an integral virus, which was used to transfer the POGLUT1 gene into BT474 human breast cancer cells in order to investigate the function and mechanism of POGLUT1.

To identify the activity of the pBabe-puro-POGLUT1-Myc recombinant retrovirus, it was transformed into 293T human embryo kidney package cells. The live retrovirus was then collected and cultured with the BT474 target cells for 48 h and POGLUT1 expression was detected. fqPCR analysis revealed high POGLUT1 mRNA expression in the BT474 cells infected with the pBaPM virus and low POGLUT1 expression in the control BT474 cells. Furthermore, western blot analysis revealed high POGLUT1 protein expression in the infected BT474 cells. These findings demonstrate that a pBaPM retrovirus with biological activity was successfully generated and could be used in the subsequent analyses.

In the present study, POGLUT1 overexpression was found to promote BT474 cell proliferation. A previous study induced the recombinant plasmid pcDNA3.1-POGLUT1 into BT474 cells with persistent TGF-β1 in the supernatant and found that POGLUT1 overexpression promoted BT474 cell proliferation detected using MTT assay (5). In the present study, BT474 cells were infected with the pBaPM recombinant retrovirus and a significant increase in cell proliferation was also observed (P<0.01). These findings suggest that POGLUT1 overexpression promotes BT474 cell proliferation.

TGF-βs are negative regulators of cell proliferation. TGF-β receptor II (TβRII) is a constitutively active protein kinase that is autophosphorylated upon TGF-β ligand binding. Phosphorylated TβRII propagates the signal through phosphorylating receptor-regulated Smad proteins, inducing their accumulation in the nucleus where they participate in the transcriptional regulation of target genes and anti-oncogenes in order to inhibit cell proliferation (24–26). In the present study, a cell growth curve was generated and showed that BT474 cell growth increased in the absence of TGF-β1, compared with in the presence of it. Furthermore, the growth curve showed that the BT474 cells overexpressing POGLUT1 rapidly grew when treated with TGF-β1 compared with the blank control cells. These findings show that POGLUT1 overexpression promotes BT474 cell proliferation.

During the regulation of the cell cycle, there are two essential checkpoints at G_1_/S and G_2_/M phase. The present study aimed to investigate which checkpoint is targeted by POGLUT1 in order to promote BT474 cell proliferation. Variations in the cell cycle were assessed in BT474 cells overexpressing POGLUT1. In total, 81.39% of the BT474 cells were found to stay in G_0_/G_1_ phase in the presence of TGF-β1 and 14.57% were found to stay in S phase, which was lower than the percentage in the control group (19.95%). However, the percentage of POGLUT1-overexpressing BT474 cells in S phase was found to be 25.80%, which was higher than that in the control group (14.57%). No significant differences were observed in any of the other cell cycle phases. These findings suggest that POGLUT1 functions primarily at the G_1_/S-phase of the cell cycle.

In the present study, POGLUT1 overexpression was found to inhibit the upregulation of the CDKI p16 by TGF-β1, as p16 expression was significantly reduced in the presence of TGF-β1 in the infected BT474 cells compared with the control cells (P<0.01). The molecular mechanism of cell cycle regulation involves cell cycle proteins (cyclins), enzymes which are activated by cyclins (CDKs) and CDK suppression proteins(CDKIs), which affect the expression and regulation of CDKs. During different phases, cyclins and their corresponding CDKs combine to form cyclin-CDK complexes, which leads to the activation of CDKs (26–28). A number of CDK suppression proteins compete with cyclins to bind to CDKs or cyclin-CDK complexes, inhibiting CDK activity. The first key step in the cell cycle is the initiation of G_1_ phase. Thus, much research has focused on the G_1_/S phase. In the G_1_ phase, cyclin D and CDK4 combine to activate CDK4, which causes retinoblastoma (Rb)-sensitive proteins to become phosphorylated resulting in the loss of the suppression of the E2F transcription factor, which may initiate DNA synthesis to induce cell cycle progression from G_1_ into S phase (29–32).

The overexpression of POGLUT1 in BT474 cells may counteract the inhibition of TGF-β1 to promote cell proliferation, suggesting that POGLUT1 may be a potential factor in the early stage of abnormal hemopoietic stem cell differentiation. Thus, further investigations are required regarding the association between the CDKIs p15 and p16, and POGLUT1.

The Taqman probe method of fqPCR analysis was used to detect p15 and p16 expression with β-actin as an internal control probe. p16 expression was observed to increase in the presence of TGF-β1, while exogenous POGLUT1 overexpression markedly suppressed p16 expression in the presence of TGF-β1. However, there was no detectable p15 expression in the BT474 cells with or without exogenous POGLUT1 expression.

These findings demonstrate that TGF-β significantly enhances p16 expression in BT474 cells and that POGLUT1 overexpression significantly reduces p16 expression in BT474 cells in the presence of TGF-β1 (P<0.01). In addition, these findings suggest that no detectable p15 is expressed in BT474 cells. For further confirmation, western blot analysis was used to assess p15 and p16 protein expression, and the findings were in accordance with those from the fqPCR analysis.

The present study also aimed to investigate the effect of POGLUT1 overexpression on p-Smad3 and to analyze the target of POGLUT1 in the suppression of p16 expression. Variations in the Smad3 protein, which acts downstream in the TGF-β signaling pathway, were assessed. Western blot analysis revealed that the overexpression of POGLUT1 inhibited the expression of p-Smad3 in BT474 cells. The protein expression of Smad3 and the internal control β-actin were observed to be expressed to the same extent in the POGLUT1 overexpression group, blank plasmid group and control group. However, p-Smad3 expression increased markedly in the two control groups and decreased markedly in the POGLUT1 overexpression group. These findings demonstrate that the overexpression of POGLUT1 may inhibit the expression of p-Smad3.

The findings of the present study suggest that POGLUT1 overexpression may inhibit p16 upregulation through TGF-β1. CDKs, cyclin D and CDKIs, including p15, p16 and p27, form a dynamic equilibrium system which maintains normal cell proliferation (33). Moreover, cyclin D and CDK4 act together to activate CDK4. However, the functional products of the p15 and p16 genes, which are p15INK4b and p16INK4a, respectively, compete with CDK4/CDK6 for cyclin D in order to suppress CDK4/cyclin D or CDK6/cyclin D complex formation and block the CDK/pRb pathway to initiate G_1_-phase arrest (34). The balance of the two pathways is a key factor in cell proliferation.

In tumor cells with abnormal proliferation, the equilibrium in the G_1_ phase is impaired, with CDKIs, including p15 and p16, becoming deactivated through certain modifications. This leads to inhibition of the CDK/pRb pathway and the release excessive E2F protein, which shortens the G_1_ phase and subsequently promotes an increase in tumor cell proliferation (35). In the present study, p16 expression was detected in BT474 cells.

The TGF-β signaling pathway has a central role in cell cycle regulation. TGF-β may combine with cell membrane acceptors in order to activate Smad3 through phosphorylation, which then forms a complex with Smad2 which enters the cell nucleus with the assistance of Smad4 in order to suppress cell proliferation through blocking its target genes. Smad3 activation has a very important role in the entire signal passage (36). Studies have shown that Smad3 is one of two CDK4/6 phosphorylation substrates. In normal cells, CDK/Smad3/p16 forms a dynamic equilibrium system, through negative feedback adjustment. Moreover, CDK4 activates Smad3 through phosphorylation, then phosphorylated Smad3 suppresses Id-1 expression. Furthermore, Id-1 may suppress Ets1 and Ets2 expression, which are located upstream of p16 and promote transcription. Thus the suppression of Id-1 increases p16 expression (35). The increase in p16 expression upon CDK4/6 inhibition decreases CDK4/6-induced Smad3 phosphorylation and the upregulation of p16 reduces through negative feedback activity (37), maintaining the dynamic balance. However, in the presence of exogenous TGF-β1, Smad3 phosphorylation no longer relies on CDK4/6 activity and the CDK/Smad3/p16 equilibrium is impaired. Exogenous TGF-β1 may strengthen the phosphorylation function of Smad3, then upregulate the expression of p16, as well as increase the inhibition of p16 on CDK4/6. Thus, cell proliferation is inhibited as the cells remain in the G_1_ phase.

The findings of the present study suggest that p-Smad3 expression is reduced in BT474 cells in the absence of TGF-β1, and that POGLUT1 overexpression reduces the endogenous phosphorylation of the Smad3 protein in order to decrease p16 expression at the gene and protein levels. In the presence of TGF-β1, p-Smad3 expression was found to markedly increase (P<0.05) and the expression of p16 also increased at the gene and protein levels. POGLUT1 overexpression was observed to decrease the phosphorylation of Smad3 and the expression of p16 was found to decrease to a level even lower than the background level, as the normal negative feedback regulation mechanism is already impaired in tumor cells. The present study found that the signaling pathway of POGLUT1 in BT474 human breast cancer cells involves a POGLUT1/Smad3/p16/CDK/pRb pathway, and the signal is increased by TGF-β1. However, further investigations on how POGLUT1 interacts with these proteins and how POGLUT1 affects Smad3 phosphorylation levels are required in order to understand the specific mechanism of POGLUT1 in cancer.

## Figures and Tables

**Table I:** BT474 cell growth following retrovirus infection.

	OD_570_			
	
Group	24 h	48 h	72 h	96 h
pBaPM retrovirus with TGF-β1	0.22±0.04	0.30±0.05	0.83±0.11^a^	0.89±0.13^a^
Blank plasmid without TGF-β1	0.19±0.03	0.29±0.07	0.75±0.11^a^	0.85±0.15^a^
Blank plasmid with TGF-β1	0.22±0.04	0.27±0.06	0.51±0.09	0.70±0.11

a P<0.01, vs. the blank plasmid with TGF-β1 group.

OD_570_, optical density at 570 nm; TGF-β1, transforming growth factor β1; pBaPM, pBabe-POGLUT1-myc.

**Table II:** Detection of p16 expression using TaqMan probes.

Groups	Ct-p16	Ct-GAPDH	ΔCt	2-ΔCt	ΔΔCt	RQ=2-ΔΔCt
POGLUT1	18.29±2.32	20.68±2.24	−2.39	5.23^a^	0.92	0.53^a^
POGLUT1+TGF-β1	25.80±3.07	25.10±2.98	0.70	0.61^b^	4.02	0.062^b^
TGF-β1	17.52±2.54	23.07±2.61	−5.55	46.83^b^	−2.24	4.72^b^
Control	18.01±3.18	21.32±3.25	−3.31	9.92	0.00	1.00

a P<0.05 and

b P<0.01, vs. the control group.

TGF-β1, transforming growth factor β1; Ct, cycle threshold; RQ, relative quantity; POGLUT1, protein O-glucosyltransferase
